# GGSYOLOv5: Flame recognition method in complex scenes based on deep learning

**DOI:** 10.1371/journal.pone.0317990

**Published:** 2025-01-31

**Authors:** Fucai Sun, Liping Du, Yantao Dai

**Affiliations:** School of Mechatronic Engineering, Harbin Vocational & Technical University, Harbin, Heilongjiang, People’s Republic of China; Jiangsu Open University, CHINA

## Abstract

The continuous development of the field of artificial intelligence, not only makes people’s lives more convenient but also plays a role in the supervision and protection of people’s lives and property safety. News of the fire is not uncommon, and fire has become the biggest hidden danger threatening the safety of public life and property. In this paper, a deep learning-based flame recognition method for complex scenes, GGSYOLOv5, is proposed. Firstly, a global attention mechanism (GAM) was added to the CSP1 module in the backbone part of the YOLOv5 network, and then a parameterless attention mechanism was added to the feature fusion part. Finally, packet random convolution (GSConv) was used to replace the original convolution at the output end. A large number of experiments show that the detection accuracy rate is 4.46% higher than the original algorithm, and the FPS is as high as 64.3, which can meet the real-time requirements. Moreover, the algorithm is deployed in the Jetson Nano embedded development board to build the flame detection system.

## 1. Introduction

**Fig 1:**
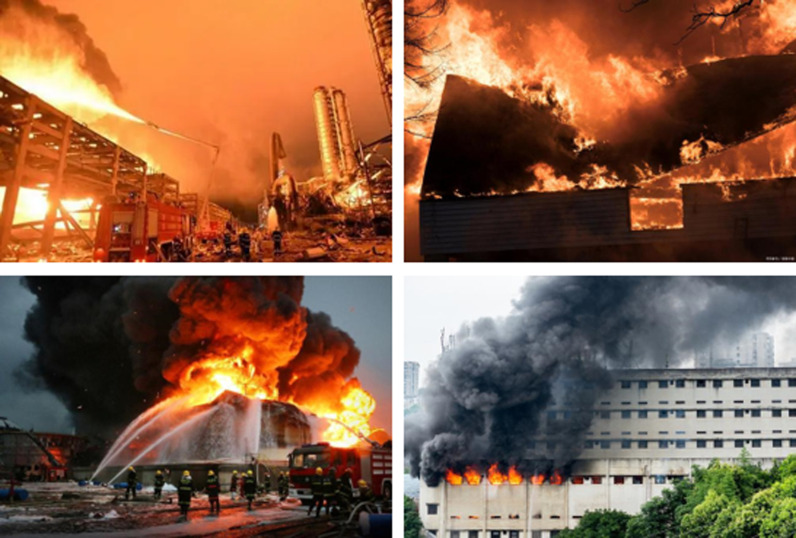
Major fire scene.

With the acceleration of urbanization and the improvement of people’s living standards, the incidence of fire accidents also increases [1]. The traditional fire monitoring method often relies on manual observation and alarm systems, but this method has many limitations, such as limited monitoring range, high false alarm, and false alarm rate, and cannot realize real-time monitoring. The fire identification technology uses computer vision, artificial intelligence, and other technologies to realize the automatic identification and alarm of the fire by analyzing the characteristics of the flame and smoke in the video image [2]. The application of this technology can greatly improve the efficiency and accuracy of fire monitoring and reduce the loss caused by fire. In addition, fire identification technology can also be applied to the field of public safety, such as airports, stations, shopping malls, and other crowded places [3,4], as shown in Fig 1. In these places, fire identification technology can monitor whether there is abnormal smoke or flames in real time, and issue alarms in time to protect people’s lives and property safety [5]. At present, there are still many defects in the fire monitoring system:

(1) The fire monitoring system must meet the real-time, need to find the fire in time, and quickly identify the location of the fire so that the security personnel can take action quickly, so as to effectively control the spread of the fire and reduce the loss caused by the fire.
(2) By analyzing image and video data, the fire monitoring system can identify fire characteristics such as flame, smoke and spark, with a high degree of accuracy and reliability, and can avoid the occurrence of false alarms and missed alarms. The fire monitoring system can automatically identify abnormal fire signs in the monitoring area, and immediately capture and archive.

The field of deep learning has continued to mature, and more and more object detection algorithms have entered our field of vision [6]. Compared with the traditional target detection algorithm, the method based on deep learning has more advantages, its reasoning speed and detection accuracy can reach a satisfactory level, its robustness is higher, and it can handle and analyze more complex situations [7]. Object detection methods based on deep learning are generally divided into two types: one-stage and two-stage object detection methods. The two-stage target detection method has higher detection accuracy, but its reasoning speed is slow, and it can’t meet the real-time requirement. Two-stage algorithms are the forerunner of deep learning-based object detection algorithms, also known as region proposal-based object detection [8]. A representative two-stage algorithm is Faster R-CNN proposed by REN et al in 2016 [9], which has relatively complex detection and training processes. The detection effect is poor, and many small objects cannot be detected. The basic principle of the one-stage object detection algorithm is to transform the object detection problem into a regression problem, obtain a prediction box through a series of calculations, and calculate the category and position of the predicted object in the prediction box, without generating a candidate box, and directly deal with the problem of the location of the target box. The one-stage target detection algorithm has the advantage in speed, but compared with the two-stage algorithm, its accuracy and accuracy may be slightly inferior [10]. However, the one-stage object detection algorithm can support multi-task learning, and can detect different objects, locate and classify target objects in the same network, which is difficult to achieve the two-stage object detection algorithm. In addition, the one-stage object detection algorithm is also suitable for restricted environments, such as smartphones and small smart devices, because it can reduce the consumption of computing resources. In terms of real-time performance, one-stage object detection algorithm is more efficient in detection. Duan et al. proposed CenterNet algorithm, which is an anchor-free target detection algorithm with advantages of both speed and accuracy [11]. Redmon and Farhadi proposed the YOLOv3 algorithm, which has low network complexity and a relatively small network model, and is very suitable for deployment on mobile devices [12]. Bochkovskiy et al. proposed the YOLOv4 algorithm in 2020 [13]. In 2020, YOLOv5 algorithm was born, showing advantages in detection accuracy and reasoning speed. Therefore, this paper chooses YOLOv5 as the benchmark. This paper improves the YOLOv5 algorithm and proposes GGSYOLOv5 algorithm. Firstly, the GAM module is embedded in the CSP1 module of CSPDarknet to improve the feature extraction capability of the network. Secondly, the no-parameter attention mechanism is added to the Neck part of YOLOv5. Finally, the convolution at the output end is replaced by a lighter GSConv. The improved algorithm has higher precision, faster inference speed, and the number of network parameters is reduced, so it is more suitable for deployment in hardware devices. The main contributions of this paper are as follows:

(1) The GAM module is embedded in each CSP1 module in CSPDarknet, and the CSP1_GAM module is proposed, which can improve the capability of network feature extraction and reduce the loss of detailed features.
(2) The SimAM parameter-free attention mechanism is added to the feature fusion part of the network to better fuse the feature information of different scales, thus improving the ability of the network to identify the target.
(3) GSConv is used to replace the common convolution at the output, which reduces the number of network parameters and effectively improves the speed of network inference.
(4) The GGSYOLOv5 algorithm model was converted into ONNX format and deployed in Jetson Nano to build the flame detection system.

The rest of this paper is organized as follows: The second section introduces the main research methods of this paper. In the third section, the ablation experiment and comparison test are carried out, and the experimental results are shown. In the fourth section, the content of the paper is discussed, and in the fifth part, the full text is summarized.

## 2. Methods

### 2.1 Global attention mechanism

Global attention mechanisms (GAM) are a core component of many modern deep learning models. It allows the model to focus on different parts of the input at different times, assigning different weights to different elements based on their relevance to the task at hand. The mechanism works by calculating the attention score for each element of the input based on how well it matches the current query. These scores are then used to create a weighted sum of the input elements, focusing the model’s attention on the most relevant parts.

The GAM attention mechanism in image processing can help the model to capture the information of different positions in the image better, and it plays an important role in processing the input image of variable length. In the field of image processing, the global attention mechanism can be applied in the following aspects: (1) In the direction of image classification: In the task of image classification, the global attention mechanism can help the model to pay global attention to the entire image, so as to better capture the feature information of different locations in the image. This helps to improve the image classification model’s understanding and classification accuracy of input images. (2) Object detection: In object detection tasks, the global attention mechanism can help the model focus on different positions in the entire image, so as to better locate and recognize objects in the image. This helps to improve the ability of object detection model to accurately detect objects in complex scenes. (3) Object detection: In object detection tasks, the global attention mechanism can help the model focus on different positions in the entire image, so as to better locate and recognize objects in the image. This helps to improve the ability of object detection model to accurately detect objects in complex scenes. Through the global attention mechanism, the model can better capture the information of different positions in the image, thus improving the performance of the image processing task. To sum up, GAM is a powerful tool in deep learning models. It allows models to dynamically allocate attention based on tasks, enhancing their ability to efficiently [14,15].

**Fig 2:**
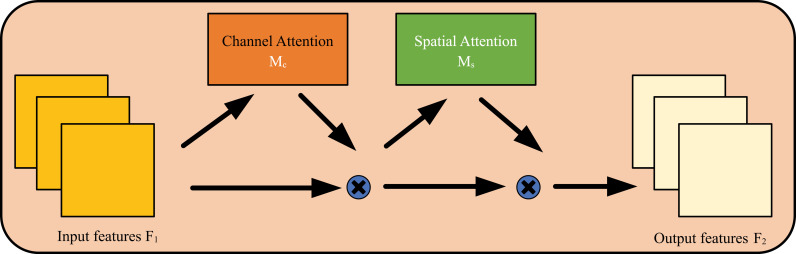
The structure of the GAM module.


The GAM attention mechanism reduces information loss and enhances global feature interaction through channel and spatial dual attention, thereby improving the performance of visual tasks. The sequential channle-space attention mechanism in CBAM is adopted, and the submodule is redesigned. As shown in Fig 2, given the input feature mapping $M_{c}$the intermediate state $F_{2}$ and output $F_{3}$ are defined as the following formula:

$$F_{2}=M_{c}(F_{1})⊗F_{1}$$ (1)

$$F_{3}=M_{s}(F_{2})⊗F_{2}$$ (2)

where $M_{c}$ and $M_{s}$ are channel and spatial attention graphs respectively

### 2.2 Group shuffle convolution

**Fig 3:**
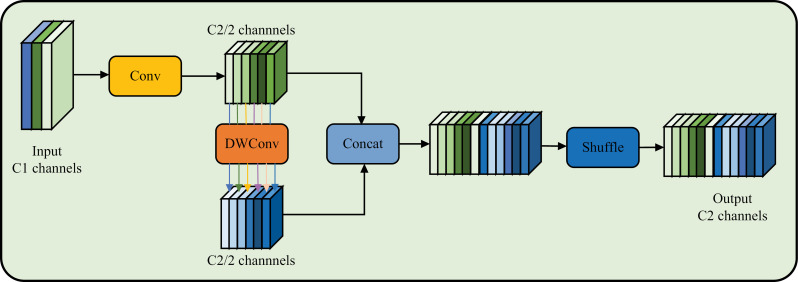
The structure of the GSConv.

Group Shuffle Convolution (GSConv) is a lightweight convolution operation, the network structure is shown in Fig 3. It is designed to enhance the efficiency and flexibility of convolution operations, particularly in scenarios where the number of channels is large. In a traditional convolution operation, all input channels are mixed and processed together. This can become computationally intensive when the number of channels is large, as it requires a significant amount of memory and computational power. GSConv solves this problem by dividing the input channels into smaller groups and dealing with each group separately. The shuffle operation within GSConv allows for more efficient memory usage and faster computation. It reduces the number of parameters required for the convolution operation, which in turn reduces the computational load and memory requirements. GSConv is particularly useful in scenarios where the number of input channels is large and memory usage is a concern. It allows for more efficient processing of high-dimensional data without significantly sacrificing accuracy. In summary, GSConv is a convolution operation that introduces a shuffle operation within the processing of input channels. It reduces memory usage and computational load, making it particularly useful in scenarios with a large number of input channels [16], allowing for a better balance between model accuracy and speed.

### 2.3 Simple attention mechanism

**Fig 4:**
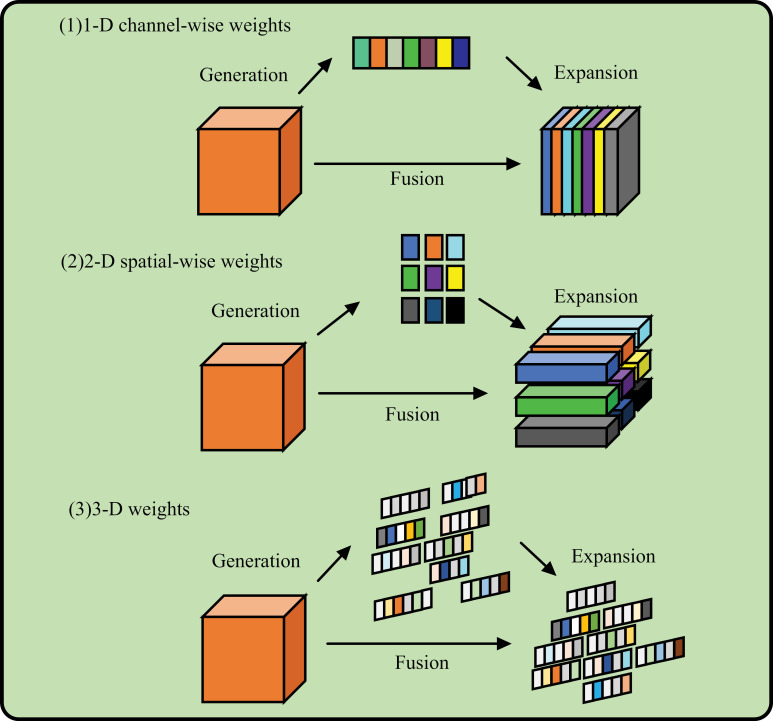
The structure of the SimAM.

The Simple Attention Mechanism (SimAM) can effectively improve model performance. It allows a model to focus on different parts of the input based on their relevance to the task at hand. The attention mechanism works by assigning weights to different elements of the input based on their relevance to the current task. These weights are calculated using a scoring function, which measures the importance of each element relative to the others. The scoring function typically compares the input elements with a query vector, which represents the current context or focus of the model. In the SimAM, the attention weights are calculated independently for each pair of input elements and query vectors. This means that each input element can have a different weight depending on the query it is being compared with. The weights are then used to create a weighted sum of the input elements, emphasizing the most relevant parts and suppressing the less relevant ones. One of the key benefits of the SimAM is its ability to dynamically focus on different aspects of the input based on the task requirements. This flexibility allows models to extract important information more efficiently and accurately, particularly in scenarios where the relationships between different elements are complex. In summary, the SimAM is a fundamental tool in deep learning models, allowing them to focus on different parts of the input based on their relevance to the task. It provides a powerful and efficient way to capture important relationships and interactions within complex data, enhancing the performance and accuracy of various machine learning applications. As shown in Fig 4 (1) represents 1D attention, which treats different channels differently and treats all locations equally, and (2) represents 2D attention, which treats different locations differently and treats all channels equally. This may limit their ability to learn more discerning cues. Therefore, the 3D weight expressed by (3) is better than the traditional one - and two-dimensional weight attention [17]. The network structure is shown in Fig 4.

### 2.4 Improved YOLOv5 algorithm

In this paper, YOLOv5 is adopted as the baseline. When the YOLOv5 algorithm is used for flame detection, it will be affected by various external factors, and the size, shape and color of the flame will be different in different environments, which requires very high detection accuracy of YOLOv5 and poses certain challenges. In this paper, the GGSYOLOv5 algorithm is proposed, which not only has a certain improvement in accuracy, but also meets the real-time requirement of reasoning speed [18].

**Fig 5:**
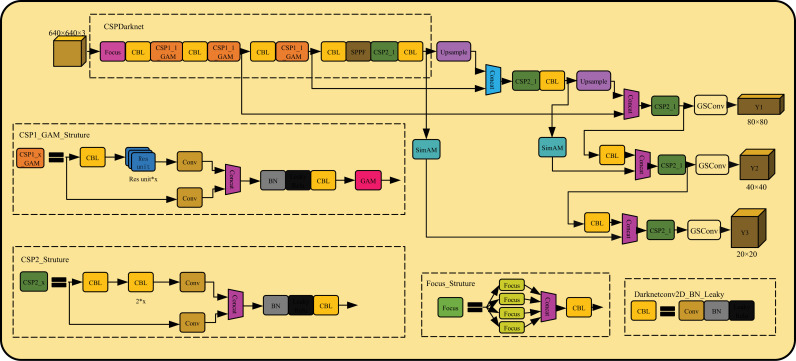
The structure of the improved YOLOv5 algorithm.

The improved YOLOv5 network structure is shown in Fig 5. The backbone network is the core of YOLOv5, which is mainly used for feature extraction of input information. The detection accuracy depends on the performance of the backbone network. Therefore, this paper adds GAM module to CSP1 module of the backbone network, and proposes CSP1_GAM module. The CSP1 structure is mainly used in the backbone of YOLOv5. The design idea is to split the input feature graph into two parts, one of which is processed by multiple residual units (such as the C3 module) to increase the network’s depth and feature extraction capabilities. The other part is simply convolution processing. Finally, the results of the two parts are concat and fused through a convolution layer. It combines the focus of GAM module on channel and space, effectively enhances the ability of network feature extraction, and improves the extraction of flame detail features in various complex scenes. Secondly, the SimAM module is introduced into the Neck part of YOLOv5 to further enhance the feature fusion capability of information of different scales. Finally, replace the original convolution with GSConv at the output. Since GSConv is a lightweight convolutional module, it can reduce the number of parameters and improve the inference speed, so that the network can detect the flame in complex scenes in real time. Compared with the original YOLOv5, GGSYOLOv5 algorithm significantly improves the detection accuracy and reasoning speed.

## 3. Experiments

### 3.1 Experimental data and evaluation index

In this paper, the pyrotechnic detection data set is used as experimental data, which includes 2,059 labeled images and 10,827 unlabeled images [19]. The data set fully demonstrates the flame picture in a variety of complex scenarios. In the training process of the network, it is far from enough to use only 2059 labeled pictures as experimental data, and a small number of pictures will cause the training to fail to achieve the ideal effect. Therefore, this paper selected 2,000 images from 10,827 unlabeled images for labeling and expanded the dataset to 4,059 images. The specific scenes and number of pictures are shown in Table 1.

**Table 1:** The number of flame pictures in various scenes.

Scene type	Number of pictures
Building fire	711
Prairie fire	624
Forest fire	564
Vehicle fires (cars, trucks, motorcycles, electric vehicles)	681
Day–night fire	823
Indoor–outdoor fire	656

Average Precision (AP) is widely used in the field of target detection to evaluate the performance of target detection algorithms. AP is a comprehensive evaluation index that takes into account changes in Recall and Precision, which is essentially the area under the PR curve. The specific formulas of Recall and Precision are as follows, which can reflect the performance of the algorithm more comprehensively [20].


$$Precision=\frac{TP}{TP+FP}$$ (3)

$$Recall=\frac{TP}{TP+FN}$$ (4)

In order to verify whether the algorithm can achieve real-time performance, it is necessary to test the inference speed of the algorithm. The inference speed of object detection can be measured in Frames Per Second (FPS). FPS represents the number of images processed per second, which can be compared under the same hardware conditions to evaluate the real-time performance of the algorithm. All experiments were model training and inference operations performed on GTX 3060 GPU.

### 3.2 Ablation experiment

Ablation experiments are commonly used in scientific research to verify the effect of a key component of a system, model, or theory on overall system performance. This paper conducted sufficient ablation experiments. This paper proposes an improvement on the basis of YOLOv5. Firstly, improvements were made in the backbone part. The module CSP1_GAM was designed to replace the original CSP1 module. The improved AP reached 63.62, which was represented by GYOLOv5 in Table 2. Secondly, the more lightweight GSConv is used as the output convolution, and the inference speed is significantly improved, the FPS is increased from the original 53.5–64.9, meeting the real-time detection effect, and it is expressed by GGYOLOv5 in Table 2. Finally, when the SimAM module was added to the feature fusion part, the AP index further increased, finally reaching 65.29, as indicated by GGSYOLOv5 in Table 2. Since all lightweight modules are used in this paper, the total number of parameters in the network is reduced.

**Table 2:** Ablation experiments result.

Method	AP (%)	FPS	Params
YOLOv5	60.83	53.5	7.332M
GYOLOv5	63.62	56.6	7.344M
GGYOLOv5	63.97	64.9	7.291M
**GGSYOLOv5**	**65.29**	**64.3**	**7.291M**

**Fig 6:**
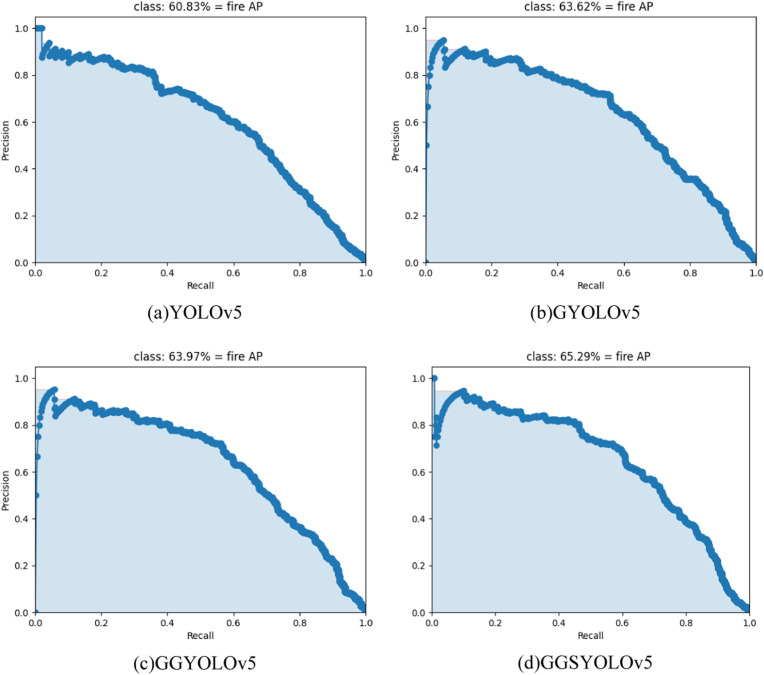
The PR curves of different models.

**Fig 7:**
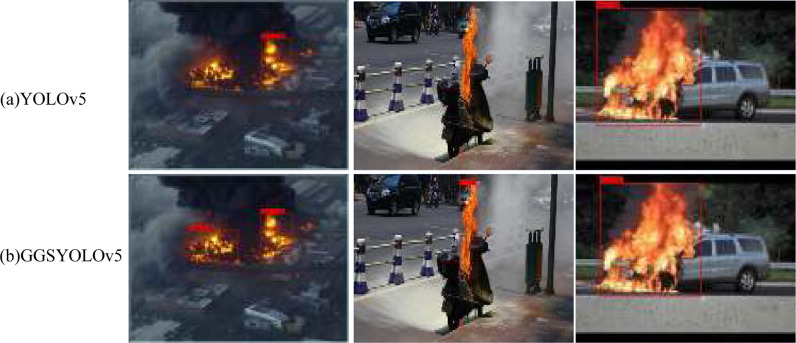
Comparison of test results between YOLOv5 and GGSYOLOv5.

Fig 6 shows the PR curves corresponding to the four models in the ablation experiment. The AP index is obtained by calculating the area under the PR curve. Therefore, the PR curve and AP index can be used to compare the difference in model detection accuracy before and after the improvement. The GGSYOLOv5 network has more advantages in accuracy. In order to more directly reflect the accuracy of GGSYOLOv5 algorithm in detecting targets, Fig 7 shows the detection results of YOLOv5 and GGSYOLOv5. GGSYOLOv5 algorithm can obviously detect more results, and the detection accuracy is higher.

### 3.3 Contrast experiment

To demonstrate the advantages of the proposed method, we compare the GGSYOLOv5 algorithm with other existing algorithms. Given that the GGSYOLOv5 algorithm is an enhancement of YOLOv5 and falls within the category of single-stage target detection, most of our comparative experiments focus on single-stage target detection algorithms. Faster R-CNN serves as a representative two-stage target detection algorithm. However, in comparison to single-stage target detection algorithms, it exhibits slower inference speed, making it unsuitable for real-time detection. On the other hand, CenterNet and the YOLO series of algorithms represent the single-stage target detection category, striking a balance between detection accuracy and inference speed, thereby meeting the requirements for real-time performance and high accuracy.

In contrast to the two-stage algorithm, CenterNet demonstrates a significant improvement in AP index and FPS. Nevertheless, a notable performance gap remains when compared to the GGSYOLOv5 algorithm. While YOLOX and YOLOv6 stand as prominent models within the YOLO algorithm series [21–23], our proposed GGSYOLOv5 algorithm exhibits superior performance in comparison. Detailed performance indicators are presented in Table 3. GGSYOLOv5 achieved the highest average accuracy (65.29) among all algorithms, followed by YOLOv6 (65.11), while Faster R-CNN achieved the lowest average accuracy (40.98). This shows that GGSYOLOv5 has a high precision in object detection. In terms of frames per second (FPS), GGSYOLOv5 also performed well, reaching 64.3 frames, slightly higher than YOLOv6 (55.7 frames) and YOLOX (53.3 frames). This means that GGSYOLOv5 can achieve high processing speed while maintaining high accuracy. In summary, GGSYOLOv5 has achieved a good balance in terms of accuracy and speed, and has certain advantages in these two indicators compared with other algorithms. Therefore, GGSYOLOv5 can be considered as one of the competitive algorithms in object detection tasks.

**Table 3:** Compare results with other methods.

Method	AP	FPS
Faster R-CNN	40.98	19.8
CenterNet	56.34	50.3
YOLOv5	60.83	53.5
YOLOX	62.91	53.3
YOLOv6	65.11	55.7
**GGSYOLOv5**	**65.29**	**64.3**

**Fig 8:**
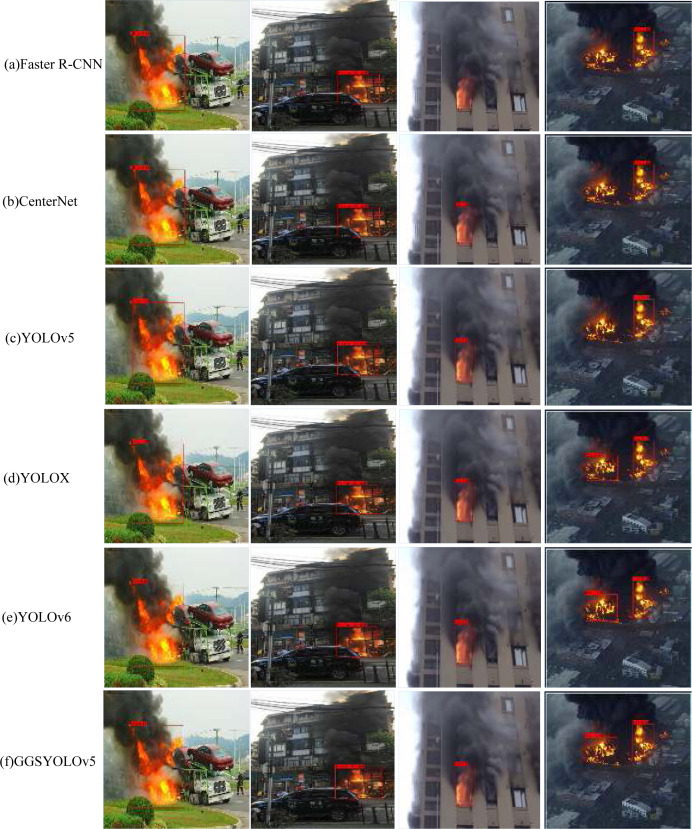
Experimental visualization results.

The visualization results of the comparison experiment are shown in Fig 8. Compared with Faster R-CNN, CenterNet, YOLO series and other algorithms, GGSYOLOv5 algorithm has higher detection accuracy and almost no missing and false detection.

### 3.4 Hardware deployment

**Fig 9:**
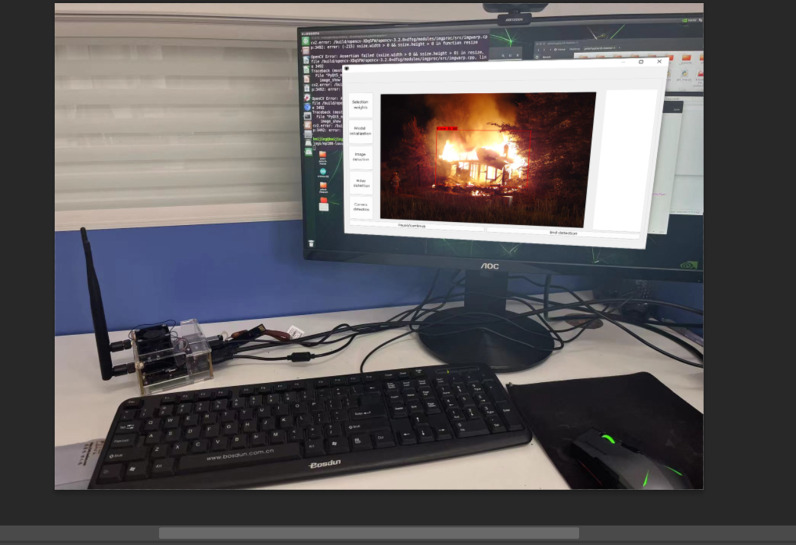
Flame detection system.

In order to put the algorithm into practical application, the GGSYOLOv5 algorithm was deployed in Jetson Nano in this paper, and the flame detection system was built by combining the display, mouse and keyboard, as shown in Fig 9. GGSYOLOv5 algorithm model is converted into ONNX format, ONNX (Open Neural Network Exchange) is an open standard for representing deep learning models. It enables different deep learning frameworks to share models, making it possible to migrate models between multiple frameworks. ONNX aims to be a bridge between different deep learning frameworks so that models can be easily shared and transformed between them.

**Fig 10:**
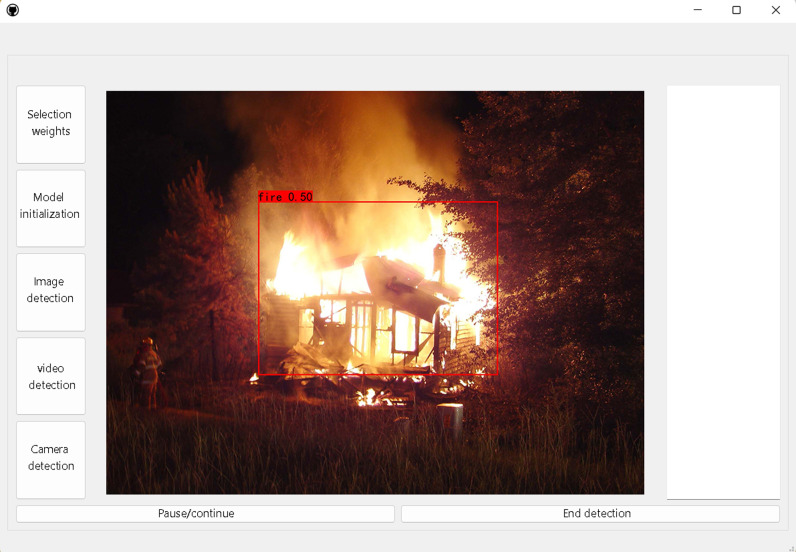
Visual interface.

For the safety of the social environment, flame monitoring is often carried out 24 hours a day. In order to make the detection process more convenient, a visual interface is designed in this paper, as shown in Fig 10, which can display the flame detection results in various scenes in real time. The specific functions of the interface are as follows:

(1) Selection weights: Select an appropriate network weight according to the requirements of the detection task.
(2) Weight initialization: After selecting the appropriate weight, the initial weight in the network, so that the network can directly call the weight in the detection process.
(3) Image detection: Open the folder and select the image you want to detect.
(4) Video detection: Open the folder and select the video you want to detect.
(5) Camera detection: Call the camera, through the external camera or computer camera for real-time monitoring.

## 4. Discussion

This paper proposes a GGSYOLOv5 algorithm for flame target detection. Compared to other methods, this algorithm demonstrates certain advantages in terms of detection accuracy, with an AP index reaching 65.29. Additionally, it offers fast reasoning speed, making it suitable for real-time detection requirements. The GGSYOLOv5 algorithm utilizes lightweight modules, ensuring that the number of parameters in the network remains unchanged compared to YOLOv5. This makes it highly suitable for deployment in hardware environments, as it is more hardware-friendly and consumes minimal computing resources. Moreover, the algorithm exhibits higher cost performance. Overall, the proposed GGSYOLOv5 algorithm not only enhances detection accuracy but also offers efficiency and compatibility with hardware environments.

## 5. Conclusion

The GGSYOLOv5 algorithm proposed in this paper has important reference value for flame target detection. Firstly, the CSP1_GAM module designed in this paper significantly improves the feature extraction capability of the backbone network. Secondly, GSConv is used instead of convolution at the output, which makes the network lighter and speeds up the reasoning speed of the network. Finally, the SimAM module is added to the Neck part of the network to improve the overall feature fusion capability of the network. Compared with other algorithms, GGSYOLOv5 algorithm has certain advantages in detection accuracy and reasoning speed. In addition, the flame detection system designed around Jetson Nano in this paper makes the GGSYOLOv5 algorithm play a role in practical applications.
